# Genomic Characterizations of Six Pigeon Paramyxovirus Type 1 Viruses Isolated from Live Bird Markets in China during 2011 to 2013

**DOI:** 10.1371/journal.pone.0124261

**Published:** 2015-04-30

**Authors:** Jingjing Wang, Hualei Liu, Wei Liu, Dongxia Zheng, Yunling Zhao, Yin Li, Yingli Wang, Shengqiang Ge, Yan Lv, Yuanyuan Zuo, Songmei Yu, Zhiliang Wang

**Affiliations:** 1 OIE Reference Laboratory for Newcastle Disease, China Animal Health and Epidemiology Center, Qingdao 266032, China; 2 China Animal Disease Control Center, Beijing 100125, China; 3 Division of Epidemiology Survey, China Animal Health and Epidemiology Center, Qingdao 266032, China; University of Maryland, UNITED STATES

## Abstract

The genomes of six pigeon paramyxovirus type 1 (PPMV-1) isolated from symptomless pigeons in live poultry markets during the national active surveillance from 2011 to 2013 were sequenced and analyzed in this study. The complete genome lengths of all isolates were 15,192 nucleotides with the gene order of 3’-NP-P-M-F-HN-L-5’. All isolates had the same motif of ^112^RRQKRF^117^ at the cleavage site of the fusion protein, which was typical of velogenic Newcastle disease virus (NDV). Several mutations were identified in the functional domains of F and HN proteins, including fusion peptide, heptad repeat region, transmembrane domains and neutralizing epitopes. Phylogenetic analysis based on sequences of complete genomes and six genes revealed that all isolates belonged to genotype VI in class II, but at least 2 sub-genotypes were identified. Most isolates were placed into sub-genotype VIb with the exception of pi/GX/1015/13, which was classified in sub-genotype VIa. The obvious antigenic difference between PPMV-1 isolates and La Sota strain was found based on the R-value calculated by cross hemagglutination inhibition (HI) assay. These results provided the evidence that PPMV-1 could be detected from healthy pigeons, and our study may be useful in designing vaccines used in pigeon, and developing molecular diagnostic tools to monitor and prevent future PPMV-1 outbreaks.

## Introduction

Newcastle disease (ND) caused by virulent Newcastle disease virus (NDV) or avian paramyxovirus type 1 (APMV-1) strain (genus *Avulavirus*, family *Paramyxoviridae*) is one of the most serious infectious diseases in poultry industry worldwide [1,2]. NDV has a negative sense, single stranded RNA genome with at least three sizes: 15,186, 15,192 and 15,198 nucleotides (nt), and contains six genes coding for the nucleocaspid protein (NP), phosphoprotein (P), matrix protein (M), fusion protein (F), haemagglutinin-neuraminidase (HN), and a large polymerase protein (L) [3,4]. NDV can be divided into class I and class II, and each class has different genotypes and sub-genotypes. Up to now, at least 18 genotypes were identified in class II [5,6,7,8]. The class I viruses with genomic size of 15,198 nt are usually avirulent to chickens and distributed worldwide in wild birds and poultry [5,9,10]. Class II viruses include some avirulent and virulent viruses. The viruses of early genotypes I-IV (originated before 1960s) possess a genome size of 15,186 nt, while viruses of recent genotypes (originated after 1960s) have a genome size of 15,192 nt [3].

Pigeon paramyxovirus type 1 (PPMV-1) is an antigenic and host variant of classical NDV of chickens, which is responsible for ND-like infectious disease of pigeons, and some strains could be distinguished from classical NDV by hemagglutination inhibition (HI) tests and monoclonal antibodies [11,12,13]. The earliest PPMV-1 isolate, designated Iraq’78, was derived from a herpesvirus stock that had been implicated in viral encephalomyelitis of pigeons in Iraqi [14,15]. Some PPMV-1 strains identified by restriction cleavage site analysis of the F gene were classified as a distinct subgroup within genotype VI (sub-genotype VIb), which were responsible for the third epizootic in the 1980s and might derive from Middle East viruses [13,16]. The complete genomic length of PPMV-1 was 15,192 nt [17], and the motif at F2/F1 cleavage site of F protein was ^112^GRQKRF^117^, ^112^RRQKRF^117^ or ^112^RRKKRF^117^, which was associated with velogenic strains, but pathogenicity index tests indicated that these isolates were of moderate or low pathogenicity for chicken, and even some had no virulence [18,19,20].

After the 1980s, PPMV-1 was still circulating worldwide [21,22,23,24]. The clinical signs observed in pigeons were closely similar to those of the neurotropic form of NDV, such as tremor of the neck and wings, torticollis, paralysis and disturbed equilibrium [25]. The respiratory signs were usually absent, only some natural infected pigeons showed respiratory signs, including gasping, coughing, sneezing and tracheal rales [21]. In this study, subclinical infection was also found in healthy pigeons obtained from live bird markets (LBMs) in China during the active surveillance. The morbidity of infected pigeons was in the range of 30–70%, and mortality was usually less than 10% [25].

In China, PPMV-1 circulated in several provinces and was recorded regularly since 1985 [26]. The viruses isolated from pigeons were placed into different genotypes, such as genotype II, VI and VII in class II [22,26]. However, in recent years, most NDVs circulating in China were isolated from chickens and belonged to class I or genotype I, II, VII, XII in class II. Therefore, most reports were focused on these epidemic strains [27,28,29], and the molecular characteristics of PPMV-1 in China are largely unknown. During 2010 to 2012, only eight PPMV-1 isolated from four provinces (Jilin, Guangdong, Liaoning and Heilongjiang) of China were sequenced and analyzed [21]. In this study, six PPMV-1 isolates obtained from different regions of China during the active surveillance from 2011 to 2013 were studied. Our results revealed similarities and differences among the six PPMV-1 strains on molecular level, and showed the antigenic difference between PPMV-1 isolates and La Sota strain, which may be useful in developing molecular diagnostic tools or designing vaccines to monitor or prevent future PPMV-1 outbreaks.

## Materials and Methods

### Ethics statement

This study was conducted according to the guidelines of animal welfare of World Organization for Animal Health, and approved by the Animal Welfare Committee of China Animal Health and Epidemiology Center (Permit number: 2013-CAHECAW-03). Swabs collected from the poultry in LBMs were approved by the owners of LBMs.

### Virus isolation and identification

**Fig 1:**
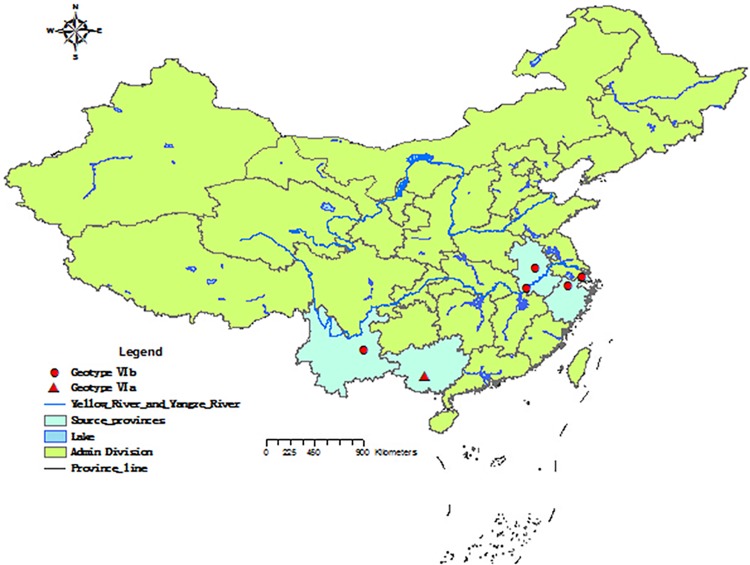
Geographical distribution of PPMV-1 strains isolated from 2011 to 2013 in China.

The PPMV-1 strains were isolated from tracheal, cloacal/fecal swabs of 67 symptomless pigeons in LBMs of one municipality (Shanghai) and four provinces (Zhejiang, Anhui, Guangxi and Yunnan) in China (Fig 1) during the active surveillance from 2011 to 2013 and propagated in 9 to 11-day-old specific-pathogen-free (SPF) eggs for 72 h. The allantoic fluid was collected and identified by standard hemagglutination (HA) and reverse transcription polymerase chain reaction (RT-PCR) assays. All isolates were plaque purified 3 passages on chicken fibroblast (DF1) cells.

### RNA extraction, RT-PCR and sequencing

Viral genomic RNA was extracted using High Pure Viral RNA Kit (Roche Applied Science) and amplified by RT-PCR with SuperScript III One-Step RT-PCR Platinum Taq HiFi (Invitrogen). Ten pairs of overlapping specific RT-PCR primers were designed based on sequences of PPMV-1 strains which were available in GenBank. For the 3’ leader and 5’trailer, primers were designed based on specific sequences of the six viruses, and the RNA was amplified using 3’-Full RACE Core Set Ver.2.0 (Takara) and 5’-Full RACE Kit (Takara) respectively according to the manufacturer’s instructions. Primer sequences used to amplify genome and 3’, 5’ ends are shown in S1 and S2 Tables. The amplified products were sequenced at Beijing Genomics Institute, China.

### Phylogenetic analysis

**Fig 2:**
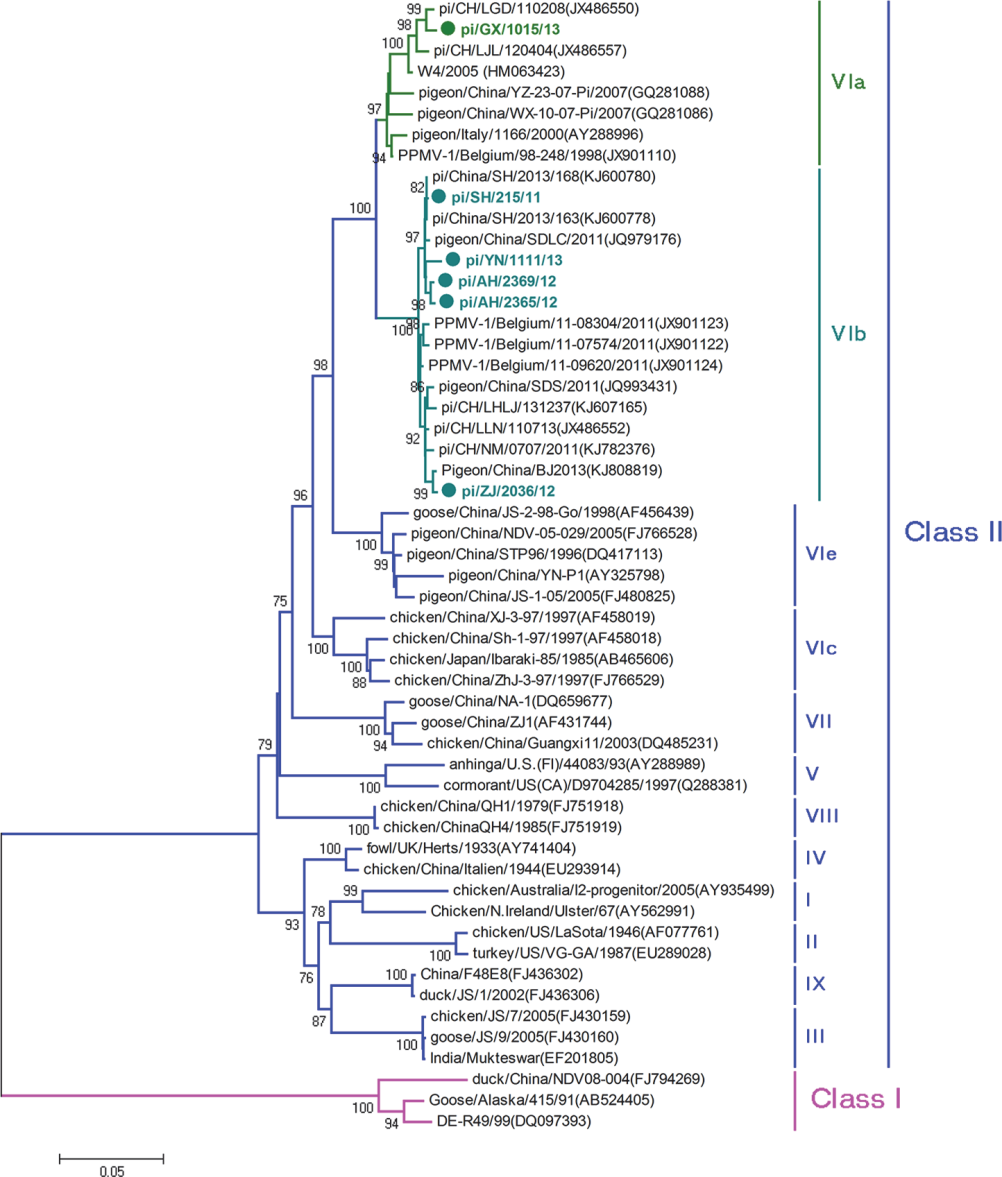
Phylogenetic analysis of six PPMV-1 isolates based on ORF (1–1653 nt) of F gene. The assembly of the matrix sequences was performed using the Clustal W algorithm in MEGA 5. The phylogenetic tree was constructed using neighbor-joining method with 500 bootstrap replicates. Sequences of previously published matrix sequences of NDV strains representing different genotypes have been included, and the genotype of each strain is indicated at the right. The GenBank accession numbers are shown in brackets. The six strains in this study are marked in bold.

The nucleotide and amino acid sequences of six isolates were assembled and aligned with homologous sequences using the Lasergene sequence analysis software package (DNAStar). The phylogenetic trees were constructed by MEGA using neighbor-joining method with 500 bootstrap replicates, and referenced the classification systems proposed by Diel and Guo, *et al* [8,21]. The 48 sequences of different genotype, which were used for phylogenetic analysis were downloaded from GenBank, and the GenBank accession numbers are shown in Fig 2.

### Determination of recombination events

Intragenic and intergenic recombination events in the complete genome of PPMV-1 strains were determined using RDP v3.44 program [30]. Seven different algorithms integrated in the program namely RDP, GeneConv, Chimaera, MaxChi, BootScan, SiScan and 3Seq (window size = 20, highest acceptable *P* value = 0.05; Bonferonni correction) were applied to estimate the occurrence of any recombination events in PPMV-1 strains.

### Cross HI assay

The cross HI assays were performed using polyclonal antisera against two PPMV-1 strains (pi/GX/1015/13 and pi/YN/1111/13) and vaccine strain La Sota. The PPMV-1 anti-serum and La Sota anti-serum used in this study were obtained from SPF chickens. Briefly, 31-day-old SPF chickens were inoculated with inactivated viruses by intramuscular route. After one month, a booster dose of each virus was administered to chickens. The sera were collected three weeks after the last inoculation and stored at -80°C until used. HI tests were conducted as described previously [31]. The titer was expressed as a reciprocal of the highest dilution of anti-serum, which caused a complete inhibition of agglutination. The antigenic relatedness of PPMV-1 isolates and La Sota was expressed in *R* value, as described by Archetti and Horsfall [32].

## Results

### Virus isolation and identification

The viruses in the allantoic fluid of inoculated SPF eggs were identified by HA and RT-PCR assays. The six viruses were designated as pigeon/Shanghai/215/2011 (pi/SH/215/11), pigeon/Anhui/2365/2012 (pi/AH/2365/12), pigeon/Anhui/2369/2012 (pi/AH/2369/12), pigeon/Zhejiang/2036/2012 (pi/ZJ/2036/12), pigeon/Yunnan/1111/2013 (pi/YN/1111/13), and pigeon/Guangxi/1015/2013 (pi/GX/1015/13). The detailed information and distribution of six isolates are shown in Table 1 and Fig 1, respectively.

**Table 1:** Related information of PPMV-1 isolates.

Isolates	Accession number	Place^a^	Time	Cleavage site
pi/SH/215/11	KM374060	Shanghai	April 2011	^112^RRQKRF^117^
pi/AH/2365/12	KM374057	Anhui	November 2012	^112^RRQKRF^117^
pi/AH/2369/12	KM374058	Anhui	November 2012	^112^RRQKRF^117^
pi/ZJ/2036/12	KM374061	Zhejiang	November 2012	^112^RRQKRF^117^
pi/YN/1111/13	KM374056	Yunnan	April 2013	^112^RRQKRF^117^
pi/GX/1015/13	KM374059	Guangxi	April 2013	^112^RRQKRF^117^

^a^ Municipality or province where specimens were collected from

### Genomic characteristics of six PPMV-1 strains

Ten overlapping fragments and sequences of 5’ and 3’ ends, covering the whole genome, were obtained using RT-PCR and assembled into one contiguous sequence. The full-length sequences of all six PPMV-1 isolates consisted of 15,192 nt, which followed the “rule of six” and the order 3’-NP-P-M-F-HN-L-5’. Compared with early genotypes of NDV (genotype I, II, III and IV), the six viruses used in this study had a 6 nt insertion (GGGGUU) in the 5’ non-coding region of NP gene between nucleotide 1,647 and 1,648. As shown in Table 2, the lengths of the 3’ leader and 5’ trailer were 55 and 114 nt respectively as reported for most NDV strains, and the 5’ untranslated regions (UTR) of six genes were always longer than 3’ UTRs. The lengths of intergenic sequence (IGS) of NP-P, P-M and M-F were 1 nt, while the IGS lengths of F-HN and HN-L were 31 nt and 47 nt, respectively.

**Table 2:** Genome length characteristics of PPMV-1 isolates.

Region	Gene sequence	3’ UTR	Coding sequence^a^	5’ UTR	Intergenic region	Nucleotide length	Amino acid length^b^
Leader	1–55					55	
NP	56–1,808	66	122–1,591	217	1	1,753	489
P	1,810–3,260	83	1,893–3,080	180	1	1,451	395
M	3,262–4,502	34	3,296–4,390	112	1	1,241	364
F	4,504–6,295	46	4,550–6,211	84	31	1,792	553
HN	6,327–8,328	91	6,418–8,133	195	47	2,002	571
L	8,376–15,078	11	8,387–15,001	77		6,703	2,204
Trailer	15,079–15,192					114	

All six isolates used in this study showed same genome length characteristics

^a^ including stop codon

^b^ without stop codon

The cleavage site of F protein in all six isolates was ^112^RRQKRF^117^, which was a characteristic of velogenic NDV. There were six potential glycosylation sites, Asn-X-Ser/Thr (N-X-S/T), in F protein, which were highly conserved in most NDVs. The major transmembrane region of six isolates was not conserved, with differences at position 502, 506, 509, 516 and 517 (Table 3). Compared with consensus amino acid sequences derived from NDV strains of different genotypes [33], the six isolates all had 2 substitutions (V121I, A132S) in fusion peptide (Table 3). Analysis of the three heptad repeat region (HR) showed 2 substitutions in HRa (143–185 aa) and 2 in HRc (471–500 aa) (Table 3). Moreover, there were 12 cysteine residues located at position 25, 76, 199, 338, 347, 362, 370, 394, 399, 401, 424 and 523 of pi/YN/1111/13 F protein, while for the other five isolates, there was an additional cysteine residue at position 27.

**Table 3:** Amino acid substitutions in the functional domains of F and HN proteins.

Virus	F protein					HN protein
	Fusion peptide 117–141	HRa 143–185	HRb 268–299	HRc 471–500	Transmembrane domain 501–521	Transmembrane domain 25–45
Consensus sequence^a^	FIGAVIGSVALGVATAAQITAAAAL	QANQNAANILRLKESIAATNEAVHEVTDGLSQLAVAVGKMQQF	LITGNPILYDSQTQLLGIQVNLPSVGNLNNMR	NNSISNALDKLAESNSKLDKVNVKLTSTSA	LITYIVLTVISLVFGALSLVL	FRIAVLLLIVMTLAISAAALV
pi/SH/215/11	V121I A132S	V179I	-	K480R	V506I V509I	I33T M35V A41V
pi/AH/2365/12	V121I A132S	V179I	-	K480R	V506I V509I	I33T M35V A41V
pi/AH/2369/12	V121I A132S	V179I	-	K494E	V506I V509I	I33T M35V A41V
pi/ZJ/2036/12	V121I A132S	V179I	-	K480R	V506I V509I	I33T M35V A41V
pi/YN/1111/13	V121I A132S	V179I	-	K480R	V506I V509I L517F	I33T M35V A41V
pi/GX/1015/13	V121I A132S	V168I	-	-	I502V V509I A516T	M35V A41V

^a^ The consensus amino acid sequence was derived from NDV strains of different genotypes

The HN genes of all six PPMV-1 isolates consisted of 2001 nt, which encoded 571 aa. In the transmembrane domain of the HN protein, pi/GX/1015/13 had 2 amino acid substitutions (M35V, A41V), while the other five isolates had 3 substitutions (I33T, M35V, A41V) (Table 3). All the six isolates had five potential glycosylation sites at position 119 (NNS), 341 (NNT), 433 (NKT), 481 (NHT) and 508 (NIS). The sialic acid binding sites and cysteine residue in six PPMV-1 isolates were completely conserved. Analysis of the neutralizing epitopes in the HN protein identified a total of six amino acid substitutions in pi/GX/1015/13, and five substitutions in other five isolates (Table 4).

**Table 4:** Amino acids constituting the neutralizing epitopes of HN protein.

Virus	HN protein									
	193–201	263	287	321	332–333	346–353	356	494	513–521	569
Vaccine strains^a^	LSGCRDHSH	N	D	K	GK/R	DEQDYQIR	K	G/D	RITRVSSSS	D
pi/SH/215/11	R197I	K	-	-	-	E347G D349E	-	-	I514V	E
pi/AH/2365/12	R197I	K	-	-	-	E347G D349E	-	-	I514V	E
pi/AH/2369/12	R197I	K	-	-	-	E347G D349E	-	-	I514V	E
pi/ZJ/2036/12	R197I	K	-	-	-	E347G D349E	-	-	I514V	E
pi/YN/1111/13	R197M	K	-	-	-	E347G D349E	-	-	I514V	E
pi/GX/1015/13	R197I	K	-	-	-	E347G D349E I352V	-	-	I514V	G

^a^ The consensus amino acid sequence of vaccine strains was derived from B1, Clone 30, La Sota, V4 and Mukteswar

The nucleotide sequence identity of genomes between pi/GX/1015/13 and other isolates was only 93.8–94.1%, while the identity of genomes among the other five isolates was 98.4–99.7%, suggesting that the five viruses shared high homology.

### Phylogenetic analysis

The six isolates were identified as genotype VI based on the phylogenetic analysis of F gene (Fig 2). Except pi/GX/1015/13, which belonged to sub-genotype VIa, the other five isolates belonged to sub-genotype VIb [8,21]. The different amino acid residues in F protein between the two sub-genotypes are shown in Table 5. The topologies of the phylogenetic trees constructed on the base of the complete genomic sequences (S1 Fig) and other 5 genes (data not shown) of PPMV-1 isolates were similar with that of phylogentic tree constructed with F gene. According to the analysis of nucleotide identity and genetic evolution, strains isolated from the same region and period had higher identities, for example, pi/AH/2365/12 shared 99.7% nucleotide sequence identity with pi/AH/2369/12. Analysis of recombination in the PPMV-1 strains using all the described methods in the RDP v3.44 program showed no recombination events (*P* < 0.05).

**Table 5:** Sub-genotype specific amino acid positions in F protein.

Sub-genotype	Virus	Amino acid positions									
		7	14	168	179	203	432	480	502	506	516
VIa	pi/GX/1015/13	T	M	I	V	T	V	K	V	V	T
	pi/CH/LGD/110208	T	M	I	V	T	V	K	V	V	T
	pi/CH/LJL/120404	T	M	I	V	T	V	K	V	V	T
	W4/2005	T	M	I	V	T	V	K	V	V	T
VIb	pi/SH/215/11	I	T	V	I	S	I	R	I	I	A
	pi/AH/2365/12	I	T	V	I	S	I	R	I	I	A
	pi/AH/2369/12	I	T	V	I	S	I	R	I	I	A
	pi/ZJ/2036/12	I	T	V	I	S	I	R	I	I	A
	pi/YN/1111/13	I	T	V	I	S	I	R	I	I	A
	pigeon/China/SDLC/2011	I	T	V	I	S	I	R	I	I	A
	pigeon/China/SDS/2011	I	T	V	I	S	I	R	I	I	A

### Cross HI assay

To evaluate the antigenic diversity of different strains, the cross HI assays were performed to calculate the *R* value. The *R* value between pi/GX/1015/13 (sub-genotype VIa) and pi/YN/1111/13 (sub-genotype VIb) was 0.71, which showed a higher similarity on antigenicity within PPMV-1 isolates even though they belonged to different sub-genotypes. However, when La Sota anti-serum was used, the HI titer using homologous antigen was higher than those using PPMV-1 strains. A comparison of La Sota strain and PPMV-1 strains (pi/GX/1015/13, pi/YN/1111/13) revealed *R* values were only 0.13 and 0.18 respectively, indicating an obvious antigenic difference between vaccine strain and PPMV-1 circulating in China.

## Discussion

PPMV-1 strains are variants of NDV associated with infections of feral and domestic (racing or show) pigeons. These viruses likely originated from Middle East and were responsible for the panzootic during the 1980s [14]. In spite of control measures, PPMV-1 infection remained enzootic in many countries and caused a degree of economic loss [21,34,35]. In this study, six PPMV-1 strains isolated from symptomless pigeons in China were genotypically characterized and were identified as genotype VI by phylogenetic analysis based on F gene.

According to the analysis of complete genome sequences, five of the six isolates were classified into sub-genotype VIb and had high homology to each other, indicating most PPMV-1 strains circulating in China mainly derived from sub-genotype VIb and there was no obvious difference among strains in distinct geographical areas. This phenomenon may be caused by migration or trade of pigeons among distant provinces in China. Even most isolates were classified into sub-genotype VIb, we also obtained one strain pi/GX/1015/13 that belonged to sub-genotype VIa [8]. This lineage derived from Middle East epizootics in the 1960s and occurred in subsequent years in Northeastern Africa and Asian countries [13]. Based on the phylogenetic analysis of genomes, pi/GX/1015/13 had a close relationship to some isolates that were obtained from pigeons in Guangdong and Jilin provinces in 2011 and 2012 respectively [21], indicating that PPMV-1 circulating in distant provinces (South China and northeastern China) were highly homologous. Another strain in the same cluster was W4/2005, which was isolated from waterbird in South China (Guangdong province) in 2005. The virus W4/2005 was deduced to be from Europe via two independent introduction events and diverged from its common ancestor at around 1999 [36]. The results of phylogenetic analysis indicated that PPMV-1 strains of sub-genotype VIa circulating in China might derive from the same ancestor with W4/2005.

The amino acid residues at F protein cleavage site have been reported to be a major determinant of pathogenicity [37,38]. The motif at F protein cleavage site of PPMV-1 was usually ^112^GRQKRF^117^, presented in PPMV-1 isolated in the 1980s, or ^112^RRQKRF^117^ that occurred in viruses obtained after the 1980s [35]. In our study, at cleavage site the six strains all possessed the motif ^112^RRQKRF^117^, which conformed to strains isolated after the 1980s and was identical to that of velogenic NDV strains. The length of HN protein, 571 aa, 577 aa, 581 aa and 616 aa, was related to viral genotype [39]. Some studies showed that the length of HN protein might contribute to virulence [17,40]. In this study, the HN length of six isolates was 571 aa, which was a common feature of most virulent NDV strains [39,41].

Comparison of functional domains of F and HN proteins between consensus sequences derived from NDV strains of different genotypes and six PPMV-1 isolates identified several amino acid substitutions. As reported, in the F protein, amino acid substitutions at fusion peptide and HR region, or replacement of transmembrane domain of NDV could affect the fusion activity of F protein [33]. In HN protein, seven substitutions were identified in neutralizing epitopes, some (N263K, E347G, I514V) of which also appeared in other NDV isolates [33]. Amino acid substitution in neutralizing epitopes was reported to play an important role in formation of antigenic epitope and could result in neutralizing escape variants [42,43,44]. Also, our results of cross HI assay showed that PPMV-1 strains had lower HI titers than La Sota strain when La Sota anti-serum was used, indicating that these amino acid substitutions in neutralizing epitopes have changed the antigenicity of PPMV-1.

Except for high mutation rate, homologous recombination also plays an important role in generating genetic diversity in RNA viruses, but there is a low frequency of recombination in negative stranded RNA viruses, especially in non-segmented negative sense RNA viruses [45,46]. For NDV, natural homologous recombination has been found in all coding genes and some non-coding regions, and the putative parents were derived from vaccine lineage and circulating virus lineage, suggesting that live vaccine strains are likely to play roles in shaping NDV evolution by homologous recombination with circulating viruses [47,48,49]. In China, the live vaccine was widely used in chickens, but rarely used in pigeons. Pigeons were mainly infected with PPMV-1 that formed a single cluster without other NDVs, which led to fewer chances for recombination of PPMV-1 and vaccine strains. Maybe this is a possible explanation for why the six viruses isolated from pigeons were not recombinants.

In summary, this study described the whole genomic characteristics of six PPMV-1 isolated from LBMs in China during 2011 to 2013. Our study indicated that PPMV-1 could produce asymptomatic infection in pigeons. So it is important to enhance the active surveillance of PPMV-1 in pigeons in case of virus shedding and further spreading. Moreover, several substitutions were observed in the functional domains of F and HN proteins, and obvious antigenic difference between PPMV-1 isolates and La Sota strain was identified by cross HI assay. These data may be useful references in designing vaccines used in pigeon and developing molecular diagnostic tools to monitor and prevent future PPMV-1 outbreaks.

## Supporting Information

S1 Table RT-PCR primers used for genome amplification. (DOCX)

S2 Table RT-PCR primers used to amplify 3’ leader and 5’ trailer. (DOCX)

S1 Fig Phylogenetic analysis of six PPMV-1 isolates based on complete genomic sequences. The assembly of the matrix sequences was performed using the Clustal W algorithm in MEGA 5. The phylogenetic tree was constructed using neighbor-joining method with 500 bootstrap replicates. The GenBank accession numbers are shown in brackets and the genotype of each strain is indicated at the right. The six strains in this study are marked in bold.
(TIF)
