# Supplementary material for: Genomic Characterizations of Six Pigeon Paramyxovirus Type 1 Viruses Isolated from Live Bird Markets in China during 2011 to 2013
Source: PLoS One. 2015 Apr 30;10(4):e0124261. doi: 10.1371/journal.pone.0124261 (PMC4415766; doi:10.1371/journal.pone.0124261)
Supplement: S1 Table — (DOCX) [file pone.0124261.s001.docx]

S1 Table. RT-PCR primers used for genome amplification

| Name | Sequence (5’→3’) | Amplified product (bp) |
| --- | --- | --- |
| NDV-P1-F | ACC AAA CAG AGA ATC TGT GAG TTA CG | 1-1209 |
| NDV-P1-R | CCG AGC CTC CAG AAT GAT GTA |  |
| NDV-P2-F | GCA TCA GTC TTG GAT AAG GGA AC | 1133-1953 |
| NDV-P2-R | CAA TGA CAG TTC CAC TGG TCT CA |  |
| NDV-P3-F | CAA CCC AAT CCA CCA ACG A | 1759-3336 |
| NDV-P3-R | GCA GCA TCA AAG TGC AGC C |  |
| NDV-P4-F | CGA AGA GAT CAG GAA GGT CAA G | 3038-4692 |
| NDV-P4-R | TTA TCT CCT GTT ACC ACA ATC CC |  |
| NDV-P5-F | CGA ATC ATC ACG ACA CCA G | 4426-6349 |
| NDV-P5-R | GCC TCT CCG ACC GTT CT |  |
| NDV-P6-F | CAG ATG AGA GCC ACT ACA AGA ACA | 6185-8216 |
| NDV-P6-R | TCC TTG GTG TTG CGA GAG ATA |  |
| NDV-P7-F | AGC ACC AAG GCA GCA TAC AC | 7978-10456 |
| NDV-P7-R | ATT GAA AGG GTC TCC TAC AAA CAT |  |
| NDV-P8-F | GAA GAG TTG CCA CTT TTA TCA CG | 10281-12056 |
| NDV-P8-R | CGG ATG ATG CCC TTA GTG C |  |
| NDV-P9-F | CAA AAT GTG ACA GCG GAG ATG | 11859-13399 |
| NDV-P9-R | AAT GGC ATC TCT GTC CTC TCG |  |
| NDV-P10-F | AGC GGA AGA GAA ATG CTC AGT A | 13216-15192 |
| NDV-P10-R | ACC AAA CAA AGA TTT GGT GAA TG |  |
