# Supplementary material for: Genomic Characterizations of Six Pigeon Paramyxovirus Type 1 Viruses Isolated from Live Bird Markets in China during 2011 to 2013
Source: PLoS One. 2015 Apr 30;10(4):e0124261. doi: 10.1371/journal.pone.0124261 (PMC4415766; doi:10.1371/journal.pone.0124261)
Supplement: S2 Table — (DOCX) [file pone.0124261.s002.docx]

S2 Table. RT-PCR primers used to amplify 3’ leader and 5’ trailer

| Name | Sequence (5’→3’) | Position (nt) |
| --- | --- | --- |
| 3’ outer | CGG AGA CAG AAT ACC GCA AA | 272-291 |
| 3’ inner | AGT GAA TAC TGG GAC CTC AAC CT | 216-238 |
| 5’ outer | TTA ATA TGC TTG ACT CGT GCT CA | 14912-14934 |
| 5’ inner | CCA TAG GTA ATG CAG CCA AGG | 14955-14975 |
