# Supplementary figures and images for: Genomic Characterizations of Six Pigeon Paramyxovirus Type 1 Viruses Isolated from Live Bird Markets in China during 2011 to 2013
Source: PLoS One. 2015 Apr 30;10(4):e0124261. doi: 10.1371/journal.pone.0124261 (PMC4415766; doi:10.1371/journal.pone.0124261)

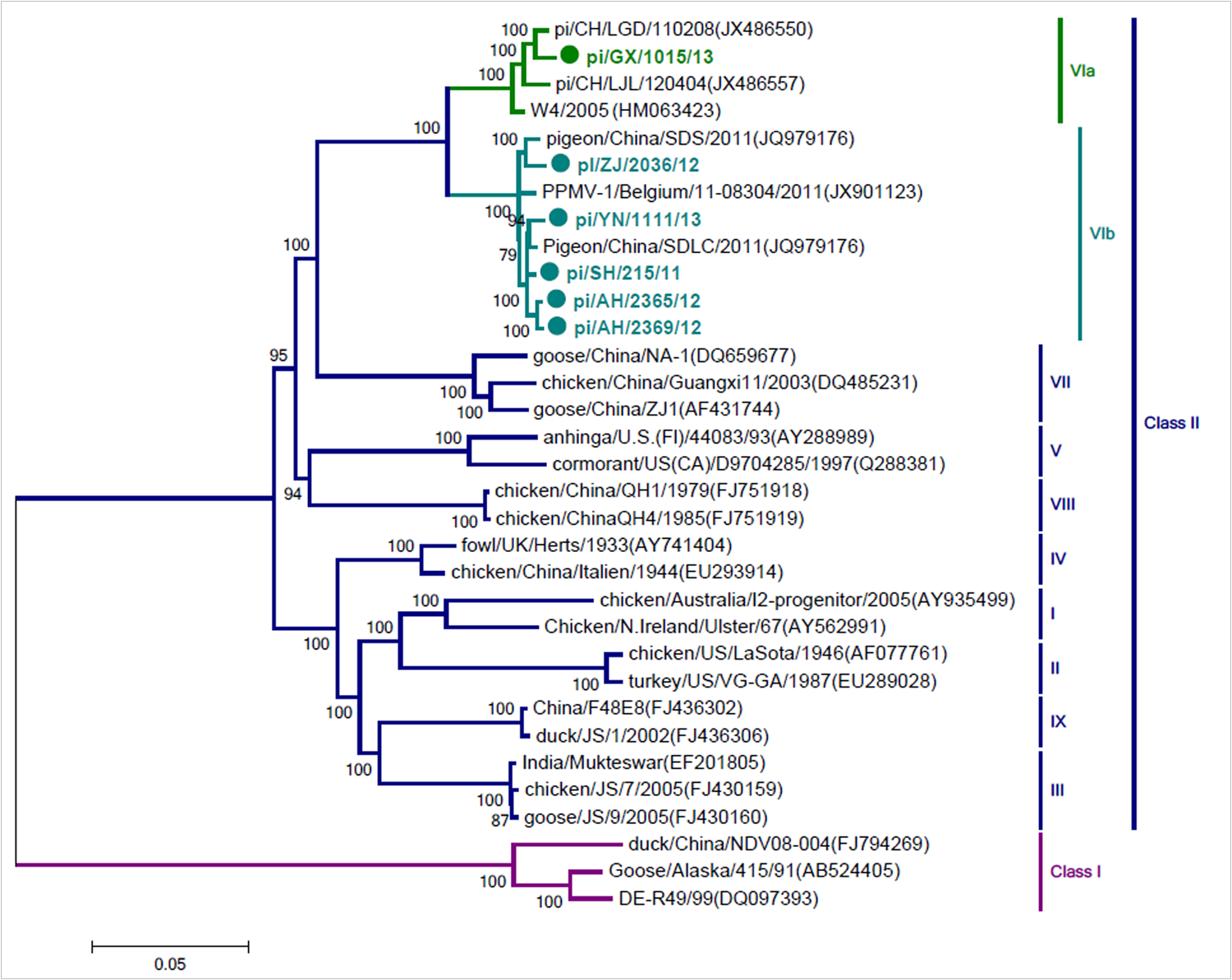

Supplement: S1 Fig — The assembly of the matrix sequences was performed using the Clustal W algorithm in MEGA 5. The phylogenetic tree was constructed using neighbor-joining method with 500 bootstrap replicates. The GenBank accession numbers are shown in brackets and the genotype of each strain is indicated at the right. The six strains in this study are marked in bold. (TIF) [file pone.0124261.s003.tif]
